# Age-related changes in the activation timing of postural muscles to the prime mover muscle for bilateral arm flexion during standing

**DOI:** 10.1186/s40101-022-00295-z

**Published:** 2022-05-07

**Authors:** Takeo Kiyota, Katsuo Fujiwara

**Affiliations:** 1 Department of Child Care and Education, Faculty of Child Care and Education, Osaka University of Comprehensive Children Education, 2-21-9 Terugaokayata, Higashisumiyoshi-Ku, Osaka, 546-0021 Japan; 2 Department of Sports Science, Kanazawa Gakuin University, 10 Sue-Machi, Kanazawa, Ishikawa 920-1392 Japan

**Keywords:** Adolescence, Anticipatory postural control, Children, Development, Electromyography

## Abstract

**Background:**

We aimed to obtain the standard values of age-related changes in the activation timing of postural muscles to the prime mover muscle (anterior deltoid [AD]) for bilateral arm flexion during standing.

**Methods:**

The study participants were 276 children (aged 3–14 years) and 32 adults (aged 20–26 years). In response to a visual stimulus, participants raised both arms from a fully extended position as quickly as possible, stopped their arms voluntarily at a horizontal level at the shoulder, and maintained that position for 2 s. Ten test trials were performed. By using surface electromyography, the duration from the burst onset of the postural muscles to that of AD was measured as the starting time of the postural muscles (rectus abdominis [RA], erector spinae [ES], rectus femoris [RF], biceps femoris [BF], tibialis anterior [TA], gastrocnemius medialis [GcM], and soleus [SOL]). The starting time was presented as a negative value when the burst onset of the postural muscles preceded that of AD, which was defined as the preceding activation. A positive value for the starting time was defined as delayed activation.

**Results:**

In adults, the burst onsets of ES and BF significantly preceded that of AD. In ES, the starting time preceded the onset of AD in those aged ≥ 5–6 years; no difference with adults was found at age 13–14 years. On the other hand, in BF, significant delayed activation was found at ages 3–4 to 11–12 years. While the starting time decreased with age, no significant preceding activation similar to adults was found, even at age 13–14 years. In TA, no significant difference with the onset of AD was found at age 3–6 years, and significant delayed activation was found at age ≥ 7–8 years. Significant delayed activation in GcM, SOL, RA, and RF was observed in all age groups, and no age-related changes were observed in children.

**Conclusion:**

These findings could provide standard values from childhood to adolescence for age-related changes in anticipatory postural muscle activity during voluntary movement while standing and contribute to applications in the fields of sports and rehabilitation.

## Background

Many studies have reported that with rapid arm movement while standing, the postural muscles of the legs and trunk that control standing posture are activated before the prime mover muscles of arm movement, which is referred to as the anticipatory activation of the postural muscles [1]. It has been presumed that this preceding activation of the postural muscles is controlled by a program selected in advance to moderate postural disturbance caused by arm movements [2]. This activation of the postural muscles is part of postural synergy as a combination of control signals sent to several muscles to ensure the stability of a limb or the whole body in anticipation of a predictable postural perturbation [3, 4]. In adults, the combination of preceding activation in the postural muscles to the prime mover muscle for arm flexion has been observed in the erector spinae (ES) and biceps femoris (BF) in posterior postural muscles during standing [5–8]. On the other hand, the triceps surae (TS) does not show apparent preceding but rather suppressed activation [5, 6, 8].

The anticipatory activation of the postural muscles during voluntary movement occurs via feedforward control, not feedback control [4]. In feedforward control, the internal model (especially the forward model), which is a reference mechanism predicting the consequence of action based on the cortical motor program and its efference copy, is essential [9]. The internal models of action are suspected to be supported by a complex network of many regions in the brain, including the prefrontal cortex, the primary and premotor cortices, the supplementary motor area, the parietal cortex, the basal ganglia, and the cerebellum [10]. It is reported that the cerebellum [11, 12] and parietal cortex [13] mainly play an essential role in the internal model. Maturation of myelination in the cerebellum has been observed in children aged 3–4 years [14]. It is also reported that the parietal cortex matures during childhood through adolescence, a process that involves significant changes in gray and white matter [15, 16]. Similar to these developmental changes in the central nervous system, it has been reported that the ability to generate motor imagery involving internal models is present by the age of 5 years, increases significantly by the age of 7–8 years, and increases further in adolescence and into adulthood [17]. Therefore, we predicted that the anticipatory activation of the postural muscles to the prime mover muscle for arm movements during standing would be changed markedly from childhood to adolescence.

In a report regarding the youngest infant observed with anticipatory activation of the postural muscles with arm movement during standing, Witherington et al. [18] demonstrated preceding activation in the gastrocnemius muscle (GcM) in a 10-month old performing the task of pulling a cabinet handle while standing. The consistency of the activation has been reported to increase with age until age 4–6 years [19], when it becomes relatively stable [20, 21]. In addition, children aged 7–9 years show preceding activation in the ES and BF with arm flexion during standing, similar to adults [22]. On the other hand, it has been reported that the development of anticipatory postural control with voluntary movement while standing is influenced by experience as well as by the development of the nervous system, resulting in significant individual differences [23]. Thus, the training effect of anticipatory postural control from childhood to adolescence has been investigated with respect to sports [24] and rehabilitation [25, 26]. However, previous studies have reported the development of anticipatory activation of the postural muscles with arm movement during standing based on typical examples or average data from a few subjects [19, 22]. In addition, these studies have not demonstrated a standard value for such development, which is the criterion for a training effect in children. Therefore, in the present study, we investigated age-related changes from childhood to adolescence in the anticipatory activation of the postural muscles with arm movement during standing in a large-scale study. The results may make it possible to obtain standard values for age-related changes in the anticipatory activation of the postural muscles, which would be a fundamental finding contributing to applications in sports and rehabilitation.

In the present study, we employed a bilateral arm flexion task during standing to investigate the anticipatory activation of the postural muscles in a large number of children and compare the findings with those in adults. The benefits of this task are that laterality for postural control does not affect the results, unlike reaching or unilateral arm flexion tasks, and younger children can understand the protocol and perform the task relatively easily. Researchers have previously examined age-related changes in postural control in children by analyzing center of pressure (CoP) displacement during an arm flexion task [27–29]. To unify the behavioral condition for each age, we adopted a simple-reaction task in response to a visual cue, which younger children can perform [28–30]. In addition, as an index of anticipatory activation of the postural muscles, we analyzed the activation timing of the postural muscles to the prime mover muscle for bilateral arm flexion during standing. In a recent study, Barlaam et al. [31] demonstrated that the timing of anticipatory activity during a bimanual load-lifting task changes with experience, resulting in enhanced postural stabilization. In children, the temporal parameter of the timing of postural muscle activity is suitable for comparison between age groups because it is difficult to compare the magnitude of the muscle activity. Therefore, by conducting these tasks and analyses, the activation of the postural muscles with bilateral arm flexion during standing can be compared among age groups.

We aimed to obtain the standard values of age-related changes in the activation timing of the postural muscles to the prime mover muscle for bilateral arm flexion during standing. The working hypothesis is as follows: The anticipatory activation timing in the ES and BF with arm movements during standing would change markedly from childhood to adolescence.

## Methods

### Participants

The study participants were 276 children (age range, 3–14 years) and 32 young adults (age range, 20–26 years). Children were divided as follows into six groups in 2-year age intervals: 48 children in the 3–4-year-old group, 69 in the 5–6-year-old group, 25 in the 7–8-year-old group, 27 in the 9–10-year-old group, 36 in the 11–12-year-old group, and 39 in the 13–14-year-old group. No participants reported having any history of neurological or orthopedic impairments. In accordance with the Declaration of Helsinki, all parents and young adults provided informed consent to participate (or for their children to participate) in the study following an explanation of the experimental protocol, which was approved by the institutional ethics committee of Kanazawa University (no. 839). The characteristics of the participants, including height, weight, and the number of individuals in each group, are shown in Table 1.

**Table 1:** Number of participants and the means and standard deviations (SDs) of physical characteristics

Age groups	Total number	Number of boys	Number of girls	Height (cm)		Weight (kg)		Foot length (cm)	
				Mean	SD	Mean	SD	Mean	SD
3–4 years	48	26	22	103.14	5.54	16.29	1.88	16.08	0.98
5–6 years	69	32	37	112.30	5.90	19.43	3.03	17.32	1.03
7–8 years	25	15	10	128.04	5.55	27.98	6.34	20.03	1.59
9–10 years	27	13	14	135.30	6.99	33.28	7.05	20.95	1.03
11–12 years	36	25	11	147.81	9.10	40.46	8.76	22.63	1.42
13–14 years	39	23	16	158.58	9.08	47.17	8.55	23.88	1.81
Young adults	32	16	16	164.78	8.49	59.97	9.66	24.02	1.63

### Apparatus and data recording

Electronic momentum signals from a force platform (OR6–6; Advanced Mechanical Technology, Inc., Watertown, MA, USA; length × width × height = 464 × 508 × 82.5 mm) were sent to an analog calculator for the CoP in the anteroposterior direction (CoP_ap_) (East Medic Co., Kanazawa, Japan) and used to determine CoP_ap_ with the following formula:

$${\mathrm{CoP}}_{\mathrm{ap}}={\mathrm{M}}_{\mathrm{y}}/{\mathrm{F}}_{\mathrm{z}}$$

where *M*_y_ is the moment in a sagittal plane and *F*_z_ is the vertical force component. A light-emitting diode (LED; diameter 5 mm) was set 1.5 m in front of the force platform at eye level and used as the ON/OFF signal indicating the beginning and end of each trial, as well as a visual target. This LED was attached to a piece of polystyrene foam accompanied by an animated character to encourage the young children aged 3–6 years to be interested in the measurement and gaze at the LED. This foam was not used for children ≥ 7–8 years and adults because they were able to fully understand the experimental procedure.

Surface electrodes (P-00-S; Ambu, Ballerup, Denmark) were used in bipolar derivation to record the electromyographic (EMG) activation of the following muscles: the anterior deltoid (AD) as the prime mover muscle for shoulder flexion, the rectus abdominis (RA) at the level of the navel, the ES at the level of the iliac crest, the rectus femoris (RF) at the midpoint between the anterior inferior iliac spine and upper border of the patella, the long head of the BF at the midpoint between the ischial tuberosity and head of the fibula, and the tibialis anterior (TA), GcM medialis, and soleus (SOL) as postural muscles. The electrode locations for the AD, TA, GcM, and SOL were the midportions of the muscle bellies. Electrodes were placed on the right side of the body with an interelectrode (center to center) distance of about 3 cm. A ground electrode was placed on the right external malleolus. These electrodes were placed after shaving and cleaning the skin with alcohol. Interelectrode impedance, as measured by an impedance tester, was reduced to below 5 kΩ. EMG signals from the electrodes were amplified (×4000) and band-pass filtered (1.6–500 Hz) using an analog amplifier (Biotop-6R12; NEC-Sanei, Tokyo, Japan) with a common mode rejection ratio of 86 dB and input impedance of > 10 MΩ.

Arm acceleration was recorded using a miniature unidirectional accelerometer (AS-5GB; Kyowa, Tokyo, Japan), which was taped to the dorsal surface of the right wrist so that the axis of sensitivity was along the sagittal plane. The vertical position of the right wrist was recorded using a position sensor system (C1373; Hamamatsu Photonics, Hamamatsu, Japan).

All electrical signals, including CoP_ap_, arm acceleration, and each EMG, were sent to a personal computer (D530; Dell Japan, Kanagawa, Japan) for analysis via an analog-to-digital converter (ADA16-32/2(CB)F; Contec, Osaka, Japan) at a 1000-Hz sampling rate and 16-bit resolution.

### Procedure

All measurements were performed on the force platform while the participants were standing barefoot with their feet 10 cm apart and parallel, elbows extended, and hands positioned on the thigh anterior to great trochanter. It is reported that the stability during quiet stance and postural disturbance is more remarkably influenced by stance width in a mediolateral direction than in an anteroposterior direction, and that mediolateral stability is increased with a stance width greater than 10 cm between both feet [32, 33]. Therefore, the stance width was set to 10 cm to eliminate the influence of mediolateral sway before the arm movement and allow focus on postural muscle activation against postural disturbance in the anteroposterior direction by arm movement.

All participants were instructed to gaze at the fixation point during all measurements. First, the participants maintained a quiet standing posture for at least 3 s. Next, in response to a visual stimulus (LED signal) randomly presented at 2–4 s after a verbal warning signal, the participants raised both arms from a fully extended position as quickly as possible, stopped their arms voluntarily at the horizontal level at the shoulder, and maintained that position for 2 s. After five practice trials, 10 test trials were performed with a 30-s rest period between each trial.

### Data analysis

All data, which were blinded to the age groups, were analyzed using signal analysis software (BIMUTAS II; Kissei Comtec, Matsumoto, Japan).

**Fig. 1:**
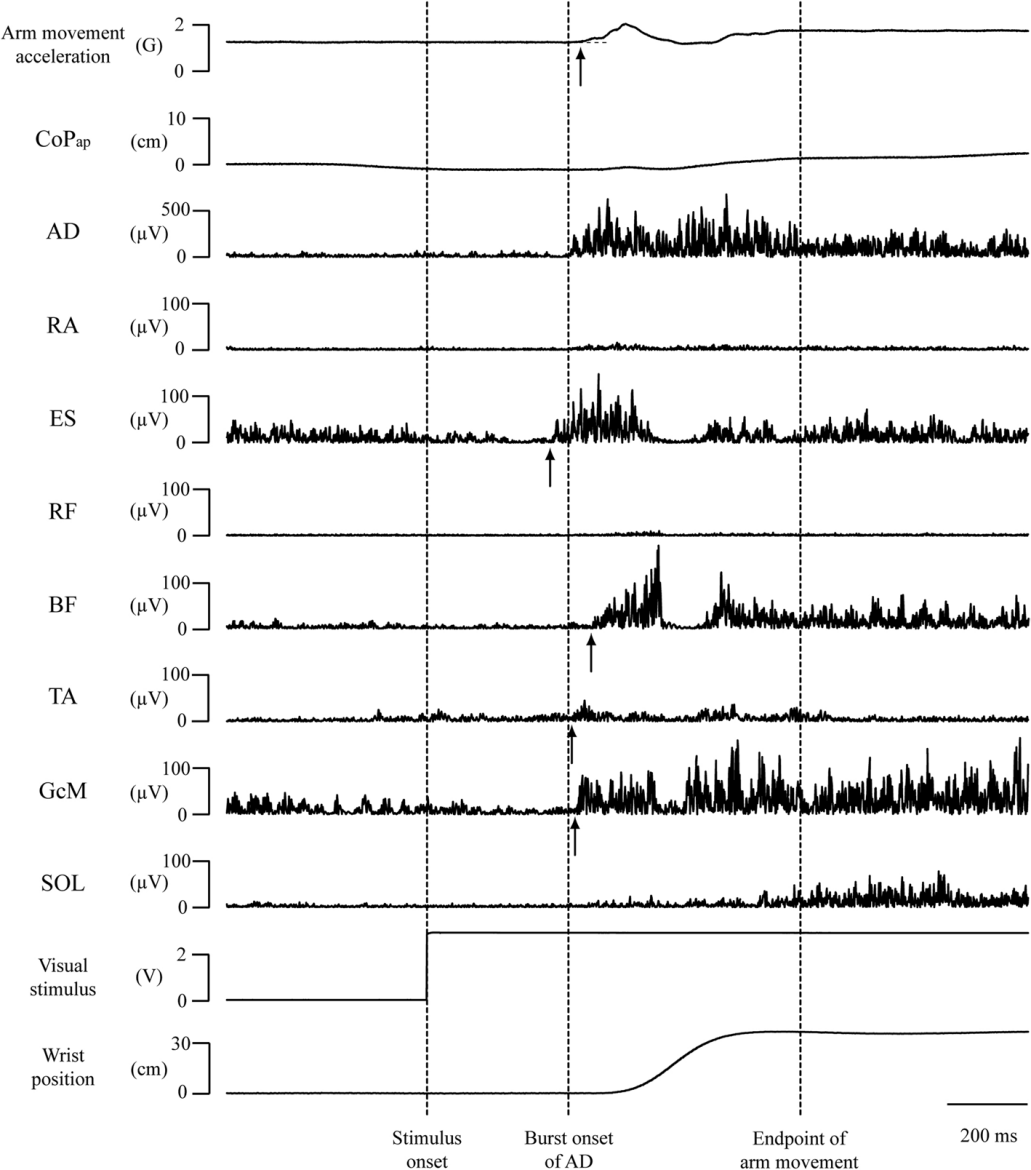
Representative electromyography waveforms for arm movement acceleration, wrist position, center of pressure in the anteroposterior direction (CoP_ap_), and visual stimulus. AD, anterior deltoid; RA, rectus abdominis; ES, erector spinae; RF, rectus femoris; BF, biceps femoris; TA, tibialis anterior; GcM, gastrocnemius medialis; SOL, soleus. Dashed lines indicate stimulus onset (left), AD burst onset (middle), and end point of arm movement (right). Straight arrows indicate burst onset of activation of the postural muscles and onset of arm movement

Arm movement duration was analyzed as described below with reference to previous studies [34, 35]. The onset of arm movement was defined as the first deviation in the accelerometer signal and the point at which the signal exceeded 5% of maximum acceleration (Fig. 1). The end of arm movement was defined as the end of the second burst activation of the AD included in the envelope line that first deviated lower than mean +2 standard deviations (SDs) from the activity calculated in the period from 500 to 400 ms before the arm lowering, with reference to the wrist position curve determined by the position sensor and acceleration curves. The interval between the starting and endpoints of arm movement was defined as the arm movement duration.

Mean CoP_ap_ positions were calculated for the periods from −300 to −150 ms with respect to the burst onset of AD (before the arm movement period) and from 0 to +150 ms to the end point of arm movement (after the arm movement period). Differences between these mean positions were defined as CoP_ap_ displacements. The mean position of CoP_ap_ and CoP_ap_ displacement was calculated as the relative distance from the heel to the total foot length (%foot length: %FL).

EMGs were analyzed as described below with reference to a previous study [34]. To exclude electrocardiographic and movement artifacts, all EMGs were high-pass filtered at 40 Hz using the seventh-order Butterworth method and then full-wave rectified.

The EMG time course was analyzed for each trial. The AD burst onset was identified by visual inspection of the EMG trace on a computer monitor since the background activation of AD before the burst onset was extremely small (Fig. 1). The time difference between the LED signal and AD burst onset was defined as the AD reaction time (ADRT). Based on studies of middle-latency stretch reflexes [36], trials in which the ADRT was below 100 ms were not considered performances of the reaction time task and thus not used in the analyses. In addition, on the basis of a study of reaction times in children [30, 37] and adults [37], ADRTs over 700 ms in children aged 3–6 years and over 500 ms in those aged ≥ 7 years were excluded from the analyses.

In previous studies, the analysis of epochs in postural muscle activation during voluntary movement was conducted as follows: (1) anticipatory postural control, the AD burst onset — 150 ms to AD burst onset; (2) compensatory postural control, the AD burst onset to the AD burst onset + 150 ms; and (3) voluntary postural control, over the AD burst onset + 150 ms [4, 38–40]. These epochs have been analyzed in the range of the AD burst onset ± reaction time with reference to the lower limit of reaction time (about 100 ms). In this study, considering individual differences in reaction time, we calculated the minimum ADRT using the following formula and set the analyzing epoch for postural muscle activation as the onset of AD ± ADRT_min_:

$${\mathrm{ADRT}}_{\mathrm{min}}={\mathrm{ADRT}}_{\mathrm{ind}}-2\times {\mathrm{ADRT}}_{\mathrm{SD}}$$

where *ADRT*_min_ is the minimum ADRT for each participant, *ADRT*_ind_ is the mean ADRT for each participant, and *ADRT*_SD_ is the SD of ADRT for each age group.

Visual inspection verified that the waves included in the envelope line of the EMG burst continued for at least 50 ms, which was within the abovementioned analyzing epoch with respect to the AD burst onset. The time point at which the wave deviated more than the mean +2 SD from the background activity was defined as the burst onset of the postural muscles. The mean and SD amplitude for the background activity of the postural muscles were calculated for the period from −300 to −150 ms with respect to the AD burst onset. The duration from the burst onset of the postural muscles to the AD burst onset was measured as the starting time of the postural muscles and presented as a negative value when the burst onset of the postural muscles preceded the AD burst onset. The activation rate of the postural muscles was calculated as the percentage of the 10 trials with burst activation. The proportion of participants with preceding and delayed postural muscle activation to the AD burst onset was calculated in each age group. “Preceding” activation was defined as when the starting time was a negative value and “delayed” activation was a positive value.

### Statistical analysis

The Shapiro–Wilk test confirmed that all data satisfied the assumption of normality. Levene’s test was used to confirm whether all data showed equal variance. One-way analysis of variance (ANOVA) was used to assess the effects of age group on starting time, the activation rate of the postural muscles, the duration of arm movement, CoP_ap_ position before arm movement, and CoP_ap_ displacement during arm movement. When a significant effect was found, a post hoc analysis was performed using Tukey’s honestly significant difference test. When equal variance was not observed, Welch’s ANOVA was applied, with the Games–Howell test used for the post hoc analysis. A Bonferroni-adjusted one-sample *t*-test was used to assess whether the burst onset of the postural muscles differed significantly from that of AD, whether the activation rate in each age group differed from the 100% rate, and whether all parameters in each age group differed from those in young adults. The chi-squared test was used to study the effect of age group on the proportion of participants with preceding and delayed postural muscle activation to the AD burst onset. Fisher’s exact test was used when an expected value in a cell was less than 5. The value of the standardized residual was used to determine what categories were major influences on a significant chi-squared test statistic. A cubic regression analysis was conducted of the starting time of the postural muscles, duration of arm movement, CoP_ap_ position before arm movement, and CoP_ap_ displacement by age. Pearson’s correlation coefficients between parameters were also calculated. The α-level was set at *p* < 0.05. All statistical analyses were performed using IBM SPSS (version 21.0J; IBM, Tokyo, Japan).

## Results

**Fig. 2:**
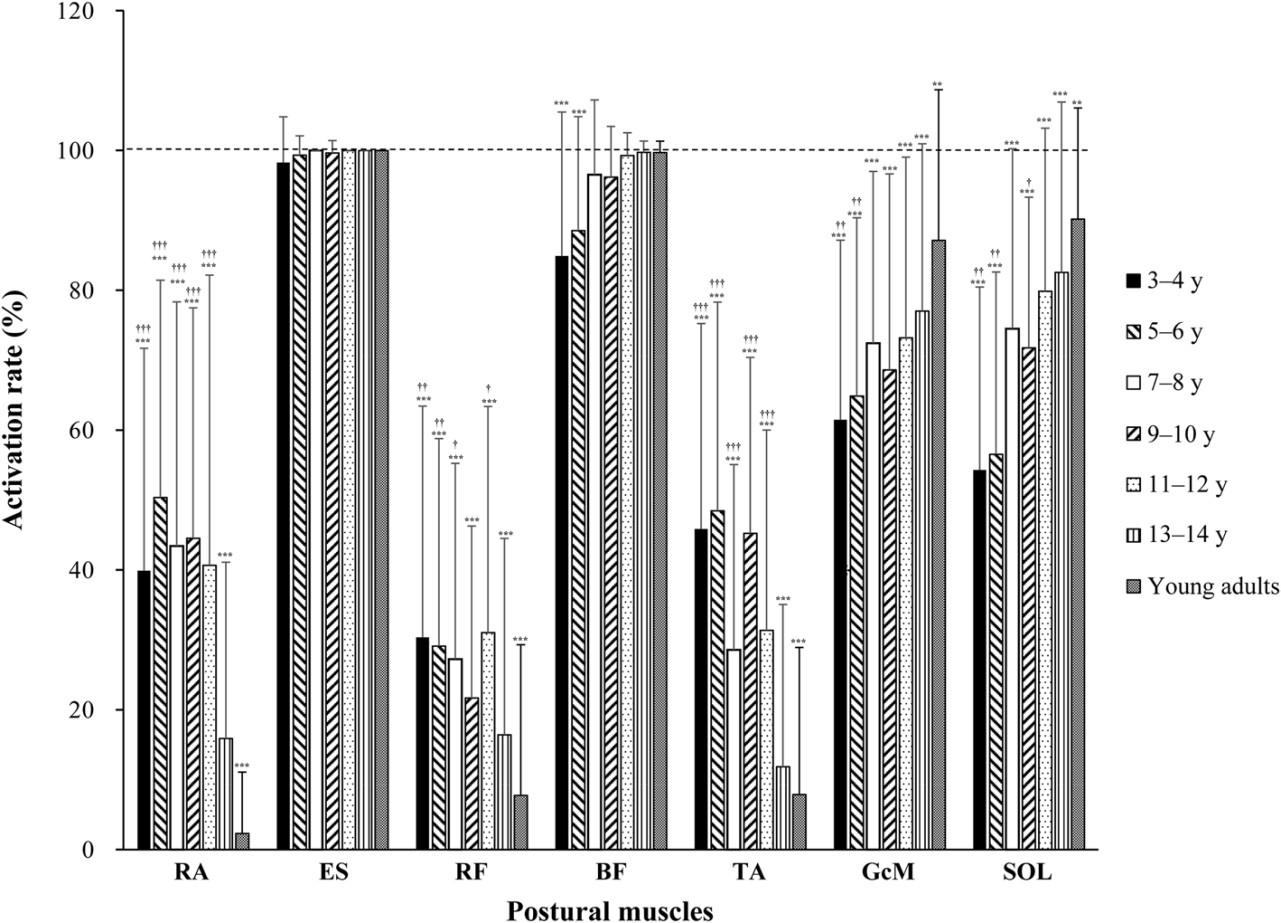
Age-related changes in the percentage of trials of postural muscles presenting burst activation (activation rate). RA, rectus abdominis; ES, erector spinae; RF, rectus femoris; BF, biceps femoris; TA, tibialis anterior; GcM, gastrocnemius medialis; SOL, soleus. Asterisks indicate significant differences relative to 100% rate. *p < 0.05, ***p < 0.001. Daggers indicate significant differences relative to adults. †*p* < 0.05, ††*p* < 0.01, †††*p* < 0.001

Age-related changes in the percentage of trials that showed burst activation (activation rate) of the postural muscles (RA, ES, RF, BF, TA, GcM, and SOL) are shown in Fig. 2. The results of the one-way ANOVA showed that age-related effects were evident in the activation rate of the following postural muscles: RA (*F*_6,101.4_ = 38.4, *p* < 0.001); RF (*F*_6,104.9_ = 4.2, *p* < 0.01); BF (*F*_6,102.4_ = 10.4, *p* < 0.001); TA (*F*_6,104.9_ = 17.7, *p* < 0.001); GcM (*F*_5,269_ = 4.5, *p* < 0.001); and SOL (*F*_6,104.9_ = 16.0, *p* < 0.001). Significant differences in the activation rates for BF, GcM, and SOL in the posterior muscles were observed between adults and children aged 4–6 years (all *p* < 0.05), but not in those aged ≥ 7–8 years. The activation rates for ES were not significantly different from 100% in all age groups or for BF in those aged ≥ 7–8 years. The activation rates for GcM and SOL were significantly smaller than 100% in all age groups (all *p* < 0.01). Regarding the anterior muscles (RA, RF, and TA), significant differences with adults were observed up until age 11–12 years (all *p* < 0.05). The activation rates for all of these anterior muscles were ≤ 20% in those aged 13–14 years and ≤ 10% in adults. The activation rates of all anterior muscles were significantly smaller than 100% in all age groups (all *p* < 0.05).

**Fig. 3:**
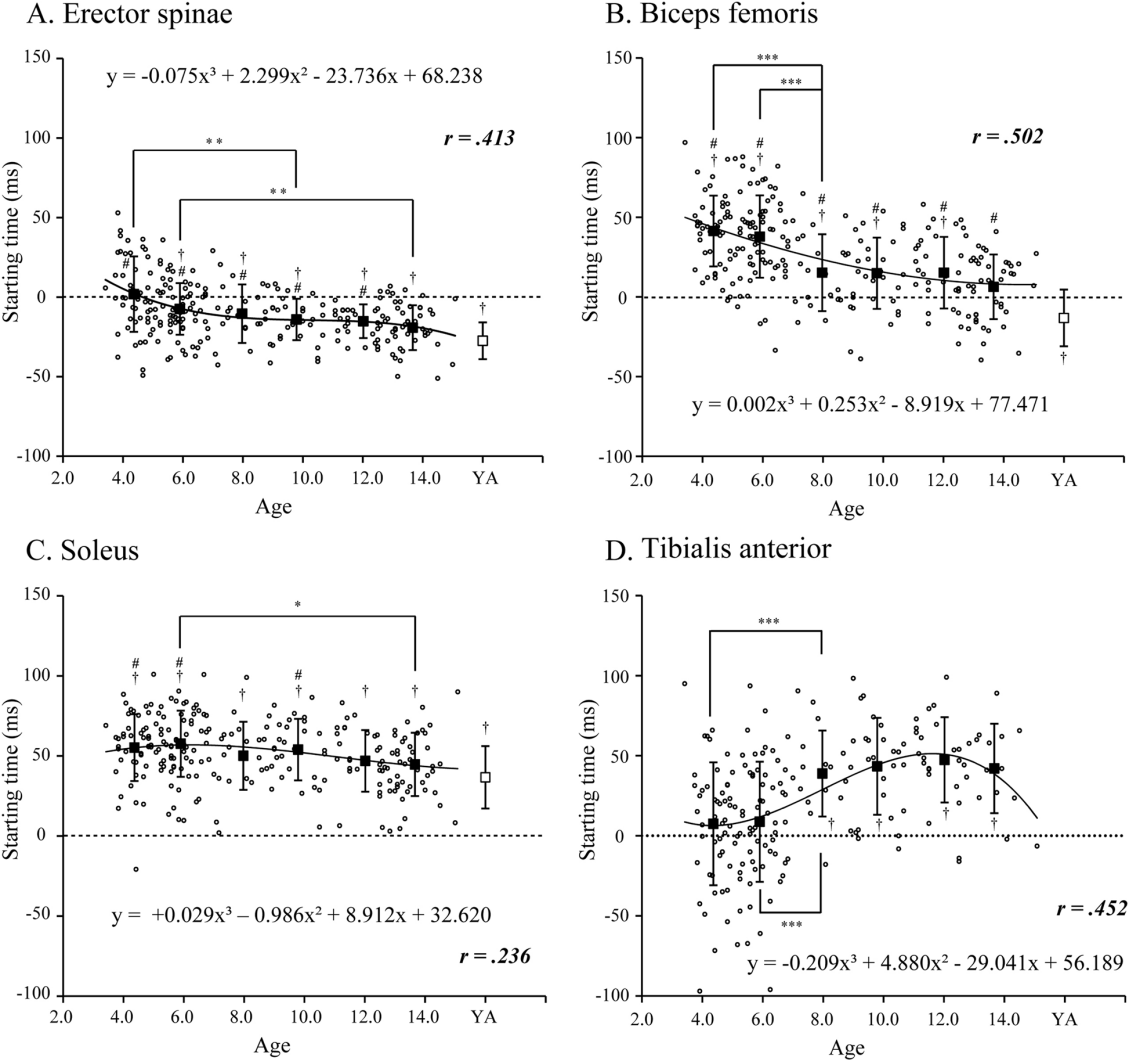
Regression analysis of the starting time of the postural muscles for age in children and mean value of the time in each age group. *p < 0.05, **p < 0.01. Small open circles indicate the starting time of the posterior postural muscles in all children aged 3–14 years. Filled squares indicate the mean starting time in each childhood age group, and open squares indicate the mean starting time in young adults (YA). The starting time for TA in adults was not included in this analysis because the activation rate was very low (< 10%). Daggers indicate significant differences relative to the burst onset of the deltoid anterior. †*p* < 0.05. Sharps indicate significant differences relative to adults. #*p* < 0.05

Age-related changes in the starting times of the posterior postural muscles (ES, BF, GcM, and SOL), which have high activation rates, are shown in Fig. 3 and Table 2. Cubic regression curves of the starting times of postural muscle activation in relation to age in 3–14-year olds showed significant changes in ES, BF, and SOL (ES, *r* = 0.41, *y* = −0.075×^3^ + 2.299×^2^−23.736× + 68.238; BF, *r* = 0.50, *y* = 0.002×^3^ + 0.253×^2^−8.919× + 77.471; SOL, *r* = 0.24, *y* = +0.029×^3^−0.986×^2^ + 8.912× + 32.620; all *p* < 0.001), with obvious age-related changes in ES (*F*_6,104.8_ = 13.15, *p* < 0.001) and BF (*F*_6,269_ = 28.60, *p* < 0.001). The ES starting time preceded the AD burst onset in those aged ≥ 5–6 years (all *p* < 0.05). The ES starting time occurred earlier with increasing age, and no differences with adults were observed in those aged 13–14 years. The BF starting time did not precede the AD burst onset at any age and was later than the AD burst onset in those aged 3–12 years (all *p* < 0.05). Although BF activation started significantly earlier from those aged 5 to 8 years (*p* < 0.05), a significant difference with adults was observed in those aged 13–14 years (*p* < 0.05). In GcM and SOL starting times, although a significant difference was observed for SOL only between those aged 5–6 and 13–14 years (*F*_6,264_ = 5.18, *p* < 0.001), these were later than the AD burst onset for all ages, and no age-related changes were observed. The cubic regression curves of the starting times of the postural muscles in relation to age showed significant changes in the anterior postural muscles (RA, RF, and TA), which have low activation rates (RA, *r* = 0.22, *y* = 0.074×^3^−2.149×^2^ + 17.946×−8.251; RF, *r* = 0.33, *y* = −0.044×^3^ + 0.078×^2^ + 8.535× + 0.874; TA, *r* = 0.45, *y* = −0.209×^3^ + 4.880×^2^−29.041× + 56.189; all *p* < 0.05; Table 2); however, a marked effect of age was observed only in TA (*F*_5,186_ = 10.23, *p* < 0.001). In TA, individual variability was considerable in those aged 3–4 and 5–6 years, showing no differences with the AD burst onset; however, a significant delay in starting time was observed in those aged ≥ 7–8 years, with activation occurring later than that of AD (Fig. 3). Although an age effect was observed for RA and RF (RA, *F*_5,177_ = 2.31, *p* < 0.05; RF, *F*_5,149_ = 2.61, *p* < 0.05), age-related changes were not observed between those aged 3–12 years who showed ≥ 20% activation rates, and activation occurred later than the AD burst onset in all of these age groups.

**Table 2:** Starting time of the postural muscles in each age groups

Groups	RA (ms)	ES (ms)	RF (ms)	BF (ms)	TA (ms)	GcM (ms)	SOL (ms)
	Mean (SD)	Mean (SD)	Mean (SD)	Mean (SD)	Mean (SD)	Mean (SD)	Mean (SD)
3–4 years	37.89 (20.99)†	1.88 (23.72)#	37.70 (33.12)†	41.40 (22.20)# †	6.46 (38.55)	46.40 (23.81)†	55.22 (20.87)†
5–6 years	33.94 (25.94)†	−7.45 (16.23)#†	42.88 (34.09)†	37.90 (25.82)#†	8.72 (37.22)	46.60 (21.75)†	57.62 (20.65)†
7–8 years	40.78 (17.51)†	−10.4 (18.39)#†	52.36 (22.40)†	15.24 (24.14)#†	39.12 (26.33)†	47.89 (21.63)†	50.11 (21.27)†
9–10 years	34.42 (20.21)†	−14.06 (13.01)#†	49.55 (23.75)†	14.89 (22.36)#†	42.97 (28.72)†	50.32 (14.23)†	53.95 (19.21)†
11–12 years	20.90 (29.57)†	−15.13 (10.60)#†	40.88 (26.21)†	15.25 (22.49)#†	46.38 (25.88)†	48.74 (21.50)†	46.93 (19.24)†
13–14 years	26.80 (14.18)†﻿	−19.21 (14.12)†	16.28 (29.66)	6.36 (20.33)#	42.05 (27.95)†﻿	49.22 (20.15)†	44.72 (19.74)†
Young adults	-	−27.44 (11.57)†	-	−13.18 (17.81)†	-	36.07 (25.23)†	36.67 (19.50)†

*RA* Rectus abdominis, *ES* Erector spinae, *RF* Rectus femoris, *BF* Biceps femoris, *TA* Tibialis anterior, *GcM* Gastrocnemius medialis, *SOL* Soleus

The starting time for RA, RF, and TA in adults was not included in this analysis because the activation rate was very low (< 10%). Daggers indicate significant differences relative to the burst onset of the deltoid anterior. †*p* < 0.05. Sharps indicate significant differences relative to adults. #*p* < 0.05

**Fig. 4:**
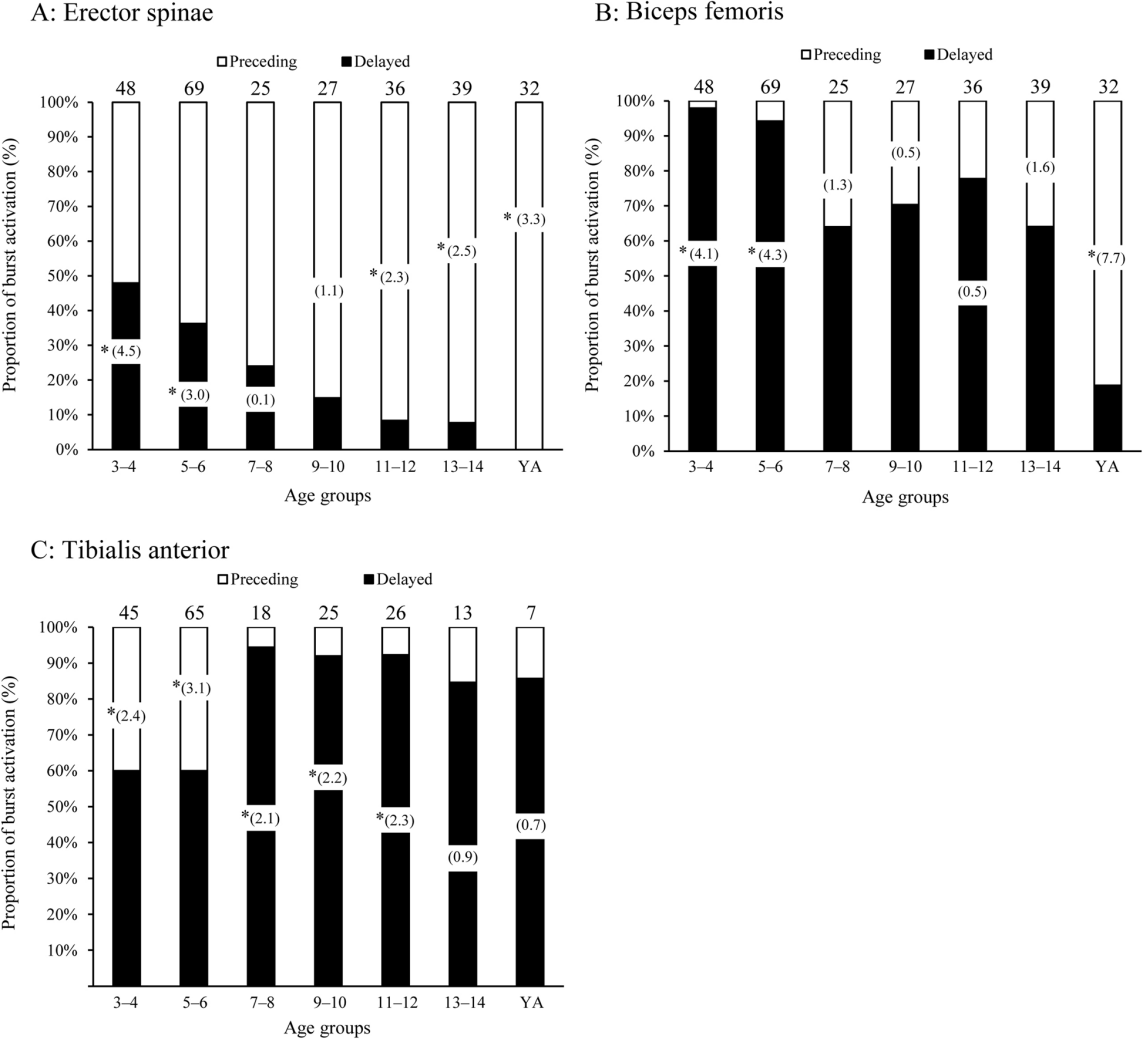
Age-related changes in the proportion of preceding and delayed burst activations. YA, young adults. Asterisks indicate a significant number of subjects relative to the expected frequency. *p < 0.05. The value over the bar graph is the number of subjects that showed burst activation in each age group

Significant age-related changes in the proportion of participants who showed preceding or delayed activation in the postural muscles to the AD burst onset were observed in only ES, BF, and TA (ES, *χ*^2^_6_ = 43.5; BF, *χ*^2^_6_ = 84.7; TA, *χ*^2^_6_ = 24.8, *ps* < 0.001; Fig. 4). Until age 6 years in ES, more participants showed “delayed” than “preceding” (standardized residuals: > 3.0). The number of participants showing “preceding” increased significantly with age; more participants showed “preceding” than “delayed” (> 2.3) in those aged > 11–12 years. Until age 6 years in BF, more participants showed “delayed” than “preceding” (> 4.1). For ages ≥ 7–8 years, the number of participants showing “preceding” increased slightly with age; more participants showed “preceding” than “delayed” (> 7.7) in adults. In TA, more participants showed “preceding” than “delayed” (> 2.4) until age 6 years and “preceding” than “delayed” (> 2.1) in those aged 7–12 years. No significant standard residuals were found in those aged 13–14 years or adults.

Age-related changes in CoP_ap_ displacement during arm flexion are shown in Table 3. The cubic regression curve of CoP_ap_ displacement in relation to age showed significant changes (*F*_3,240_ = 5.84, *r* = 0.26, *y* = −0.0004×^3^ + 0.042×^2^−0.868× + 12.236, *p* < 0.01). CoP_ap_ displacement had an age effect (*F*_5,94.9_ = 3.57, *p* < 0.01), and significant differences with those aged 3–4 years were observed in those aged 9–10 and 13–14 years (*p* < 0.05). Adults had the smallest displacement, and all child groups except those aged 9–10 years showed a significant difference with adults (all *ps* < 0.05). A correlation between the starting times of ES and CoP_ap_ displacement was found only in those aged 3–4 years (*r* = 0.56).

**Table 3:** Age-related changes in the displacement of the center of pressure in the anteroposterior direction (CoP_ap_) during arm flexion, arm movement time, and the position of CoP_ap_ before arm movement

Groups	CoP_ap_ displacement (%FL)	Movement time (ms)	CoP_ap_ position before arm movement (%FL)
	Mean (SD)	Mean (SD)	Mean (SD)
3–4 years	9.15 (3.99)#	648.24 (84.83)#	39.69 (5.08)#^c^
5–6 years	8.61 (3.19)#	615.41 (91.06)#	39.05 (6.46)#^c^
7–8 years	7.94 (2.64)#	555.13 (89.21)^a^	39.25 (5.71)#^c^
9–10 years	6.74 (3.02)^a^	568.88 (67.28)^a^	41.29 (6.82)
11–12 years	7.32 (2.04)#^a^	544.37 (69.97)^ab^	44.33 (6.30)
13–14 years	7.26 (1.84)#^a^	559.35 (35.74)^ab^	41.18 (6.82)
Young adults	5.59 (1.94)	525.96 (75.65)	44.92 (4.63)

Sharps indicate significant differences relative to adults. #*p* < 0.05. ^a^Indicates a significant difference compared to 3–4 years (*p* < 0.05). ^b^Indicates a significant difference compared to 5–6 years (*p* < 0.05). ^c^Indicates a significant difference compared to 11–12 years (*p* < 0.05). *FL* Foot length

Age-related changes in arm movement time and CoP_ap_ position before arm movement during arm flexion are shown in Table 3. Cubic regression curves for all variables in relation to age showed significant changes (arm movement time, *r* = 0.46, *y* = −0.035×^3^ + 2.839×^2^−52.229× + 827.434; CoP_ap_ position, *r* = 0.23, y = −0.028×^3^ + 0.758×^2^−6.061× + 53.956; all *ps* < 0.05), demonstrating an age effect (arm movement time, *F*_5,92.6_ = 12.4; CoP_ap_ position, *F*_3,240_ = 4.44; all *p* < 0.05). Correlations between the starting times of ES, BF, and TA and CoP_ap_ position were found in adults’ BF only (*r* = −0.53, *p* < 0.05). Also, the starting time of TA in those aged 3–6 years, which showed large individual variation, was correlated with CoP_ap_ position (*r* = 0.30, *p* < 0.05). Arm movement time was not significantly correlated with any age group or postural muscle.

## Discussion

In this study, we investigated age-related changes from childhood to adolescence in the anticipatory activation of the postural muscles with arm movement during standing in a large-scale study and obtained standard values for age-related changes. In voluntary postural control during arm movements, the activation pattern of postural muscles is known to be affected by arm movement dynamics and behavioral conditions [41]. In the present study, arm movement time was not significantly correlated with any age group or postural muscle for the arm movement dynamics. Additionally, to unify the behavioral condition in each age group, trials that deviated from the analysis range of the AD reaction time were excluded [30, 37], and participants likely performed the simple-reaction tasks regardless of age.

In adults, preceding activation to the AD burst onset was observed in ES and BF, but not in GcM and SOL; these results are consistent with previous studies [5, 6, 8]. On the other hand, the present study revealed the following in children and adolescents: (1) age-related changes in preceding activation in ES were different from those in BF, (2) the proportion of preceding activation in TA was observed in 40% of children aged 3–6 years, and (3) no preceding activation was observed in GcM and SOL.

### Age-related changes in preceding activation in ES and BF to the AD burst onset

ES showed preceding activation in children aged ≥ 5–6 years, while BF did not, even in those aged 13–14 years. The activation timing of the postural muscles during arm flexion in a simple-reaction task has been suggested to be affected by time constraints [42–44]. Therefore, anticipatory activation of the postural muscles is focused on the preparatory activation in the trunk because of the insufficient postural preparation in this task [34]. Although CoP_ap_ displacement during arm flexion was greater in children than in adults, no significant differences in CoP_ap_ displacement among children aged ≥ 5–6 years were found. In these children aged ≥ 5–6 years, CoP_ap_ position after arm flexion was within a stable range in the standing posture [45]. These results suggest that preceding activation in ES only is sufficient to reduce postural disturbance in children. In addition, development in coordinative control of the trunk and legs is reported to be insufficient in children [46]. Therefore, it is possible that the focus of anticipatory postural muscle activation in children aged ≥ 5–6 years affected by the time constraint in the simple-reaction task was in simple control of the trunk only, not in complex and coordinative control of the trunk and legs. Similarly, it has been reported that adults and children aged 10 years make compensatory trunk movements for accurate reaching while standing, while children aged 7 years stabilize the trunk to improve reaching [47]. Furthermore, the correlation between the starting times of ES and CoP_ap_ displacement was found only in those aged 3–4 years. The preceding activation in ES probably affects CoP_ap_ displacement in those aged 3–4 years. However, no significant correlations were found in those aged ≥ 5–6 years. These results could be due to the fact that almost all subjects showed preceding ES activation in those aged ≥ 5–6 years. In the present results, the difference in age-related changes in anticipatory activation of the postural muscles between age groups in children and adults was the presence or absence of preceding activation in BF. Therefore, in children aged ≥ 5–6 years, it would be presumed that though the postural disturbance shown by CoP_ap_ displacement is moderated by preceding activation in ES, it is not yet at an adult level because of the absence of preceding activation in BF.

BF activation was significantly delayed compared with AD until 3–12 years of age but showed simultaneous activation with AD at 13–14 years of age. These results suggest that age-related changes in the BF begin at 13–14 years of age, and that preceding activation of both the ES and BF as seen in adults would be found in children from age 15 years to young adulthood. In adolescence, the musculoskeletal system grows considerably. It has been reported that developmental changes in the interaction between the internal representation of the body and environment affect the development of anticipatory postural control [48, 49]. Many studies have suggested that the construction of an internal model of action continues to develop during adolescence [50, 51]. This model’s developmental changes could be based on the ongoing maturation of the central nervous system, including the parietal cortex [52, 53]. In adolescence, the internal model associated with anticipatory postural control during arm flexion during standing, including ES and BF, may continue developing in those aged 15 years.

In addition, correlations were found between the starting time of BF and CoP_ap_ position in adults only. Sensory data from the BF is an essential source of information for perception in the standing position relative to quiet standing [54]. It is presumed that information in the standing position based on the BF is used to build an internal model of anticipatory postural muscle activation.

### Age-related changes in preceding activation in TA to the AD burst onset

In TA, no significant difference with the onset of AD was observed in children aged 3–6 years, and a significant delay in starting time was observed in those aged ≥ 7–8 years. The proportion of children aged 3–6 years showing preceding activation was 40% and decreased to about 10% at age ≥ 7–8 years. The CoP_ap_ position before arm movements was more posterior in those aged 3–6 years than in the other age groups. A previous study reported that the CoP_ap_ position during quiet standing is 40% FL more posterior in children [55]. The lower leg is inclined forward by activation in the TA [56]. Therefore, preceding activation in the TA may play a role in the forward inclination of the lower limb to prevent the over-backward inclination of the whole body by preceding activation in the ES during arm flexion.

### No preceding activation in GcM and SOL

No anticipatory activation in GcM and SOL was observed in any group. In previous studies, similar patterns in adults have been reported [5, 6, 8, 22]. Since postural disturbance by arm flexion affects the trunk with a relatively high mass close to a prime mover (AD), moderating the postural disturbance for the trunk by preceding activation in ES would be necessary when arm flexion is performed from the arm hanging position. On the other hand, activation in GcM and SOL in all age groups would be less necessary in this task. Several studies have supported these findings. Preceding activation in TS was found when arm flexion with limited CoP_ap_ anterior displacement is performed to focus postural control at the ankles, even if the flexion is performed from the arm hanging position [57]. In addition, preceding activation in GcM has been reported during a handle pull task while standing, regardless of age [3, 19]. Crenna et al. [58] suggested that backward inclination of the whole body pivoting at the ankles based on the activation of TS is the postural movement pattern required to translate the center of gravity (CoG) backward effectively. These previous findings suggest that preceding activation of TS during arm flexion changes to translate CoG effectively, depending on the postural task. Since the influence of perturbations by arm flexion is relatively small, anticipatory activation in TS would be less necessary in this task for not only adults but also children.

Cordo and Nashner [3] found that the activation pattern of postural muscles associated with voluntary movement changed according to internal and external conditions, which suggests that children can select the appropriate motor strategies according to the postural conditions with development in postural control [59]. Age-related changes in the anticipatory activation pattern of postural muscles depending on external conditions, including in children aged ≥ 15 years, should be examined in detail in future studies.

## Conclusion

In this large-scale study, we identified age-related changes from childhood to adolescence in the anticipatory activation of the postural muscles with arm movement during standing. Age-related changes in activation timing of postural muscles to the prime mover muscle for arm flexion during standing differ depending on the postural muscles. We therefore report the following findings: (1) ES showed preceding activation to the AD burst onset at age ≥ 5–6 years, while BF did not, even at age 13–14 years. (2) The proportion of preceding activation in TA was 40% in children aged 3–6 years. (3) No preceding activation was found in GcM or SOL. These findings could be standard values for age-related changes from childhood to adolescence in anticipatory postural muscle activity during voluntary movement while standing and contribute to applications in the fields of sports (e.g., balance training for junior athlete) and rehabilitation (e.g., training of activity of daily living for children with cerebral palsy).

## Data Availability

Not applicable.

## Abbreviations

AD: Anterior deltoid
ANOVA: Analysis of variance
BF: Long head of the biceps femoris
CoG: Center of gravity
CoP_ap_: Center of pressure in the anteroposterior direction
EMG: Electromyography
ES: Erector spinae
FL: Foot length
Fz: Vertical force component
GcM: Gastrocnemius medialis
LED: Light-emitting diode
My: Moment in a sagittal plane
RA: Rectus abdominis
RF: Rectus femoris
RT: Reaction time
SOL: Soleus
TS: Triceps surae
